# Histopathology Caused by the Entomopathogenic Fungi, *Beauveria bassiana* and *Metarhizium anisopliae*, in the Adult Planthopper, *Peregrinus maidis*, a Maize Virus Vector

**DOI:** 10.1673/031.010.3501

**Published:** 2010-04-17

**Authors:** A.V. Toledo, A.M.M. de Remes Lenicov, C.C. López Lastra

**Affiliations:** ^1^ Centro de Investigaciones de Fitopatología (CIDEFI). Facultad de Ciencias Agrarias y Forestales, UNLP. Calle 60 y 119, (1900) La Plata, Buenos Aires, Argentina; ^2^ División Entomología. Facultad de Ciencias Naturales y Museo. UNLP. Paseo del Bosque s/n. (1900), La Plata, Buenos Aires, Argentina; ^3^ Centro de Estudios Parasitológicos y de Vectores (CEPAVE) UNLP-CONICET. Calle 2 Nro. 584 (1900) La Plata, Buenos Aires, Argentina

**Keywords:** antagonistic bacteria, pests of cereal crops

## Abstract

The planthopper *Peregrinus maidis* (Ashmead) (Hemiptera: Delphacidae) is an important vector of maize viruses in tropical and subtropical areas. Planthoppers are biologically controlled with several species of entomopathogenic fungi that have been isolated from these insect pests of rice in Asia. *Beauveria bassiana* (Balsamo-Crivelli) Vuillemin and *Metarhizium anisopliae* (Metschnikoff) Sorokin (Hypocreales: Clavicipitaceae) appear to be the most useful against planthoppers because of their ease of mass production, storage, virulence, and application. In the present study, adults of *P. maidis* infected with *B. bassiana* and *M. anisopliae* were observed under light and scanning electron microscopy to characterize morphologically the process of infection and the development of these fungi, prior to and after the death of the host. The hydrophobic conidia of both fungal species were able to attach to all body regions, with a preference for surfaces containing hairs. Few germinated conidia were observed on the insect's body surface at 24, 48, and 72 hr post-inoculation. On the cuticular surface of *P. maidis* treated with *B. bassiana* and *M. anisopliae*, bacillus-like bacteria were observed. These microorganisms could be interacting with fungal conidia, playing a role of antibiosis that will not allow the fungal pathogens to germinate and penetrate. In the colonization events observed in this study, the formation and multiplication of hyphal bodies by both fungal species inside the host's body was noted. The host's whole body was invaded by hyphae between five and six days post-inoculation, and body fat was the most affected tissue.

## Introduction

In tropical and subtropical areas, maize *Zea mays* L (Poales: Poaceae) can become infected with destructive viruses including maize stripe tenuivirus, maize mosaic rhabdovirus, maize Iranian mosaic virus, and maize sterile stunt virus. These viruses are transmitted to the host plant by the planthopper *Peregrinus maidis* (Ashmead) (Hemiptera: Delphacidae), which is broadly distributed, especially in tropical and subtropical regions (Nault and Ammar 1989). In Argentina, *P. maidis* was reported as an experimental vector of maize disease caused by Mal de Río Cuarto virus (MRCV) belonging to the *Fijivirus* genus (Virla et al. 2004). This vector was collected on maize (*Zea. mays*, and *Z. perennis*), sorghum (*Sorghum vulgare* and *S. halepense*), millet (*Panicum miliaceum*), and citrus in locations in the Formosa, Chaco, Corrientes, Entre Ríos, Jujuy, Salta, Tucumán, and Buenos Aires provinces of Argentina (Tesón and Remes Lenicov 1989; Remes Lenicov and Mariani 2001). Several entomopathogenic fungi have been isolated from planthopper pests of rice crops in Asia, and some of these have been evaluated for control of these pest insects (Aguda et al. 1987; Rombach et al. 1986a, b). Among these fungi, *Beauveria bassiana* (Balsamo-Crivelli) Vuillemin and *Metarhizium anisopliae* (Metschnikoff) Sorokin (Ascomycota: Clavicipitaceae) seem to be the most useful against these insects because of their ease of mass production, storage, virulence, and application (Rice and Choo 2000). Although some workers have investigated the modes of infection and the histopathology of these fungi in selected insects of economic importance, there are no studies referring to the histopathology of entomopathogenic fungi in planthoppers. In the present study, adults of *P. maidis* were infected with *B. bassiana* and *M. anisopliae* and studied under light and scanning electron microscopy to determine the infection processes and the development of these fungi, before and after host death.

## Materials and Methods

Planthoppers were reared on maize plants grown in plastic flowerpots and isolated inside 24cm × 9cm polyethylene terephthalate plastic cages in a greenhouse under 20 ± 5° C and a natural photoperiodicity. The *B. bassiana* strain used in this study was isolated from one adult of *Oliarus dimidiatus* Berg (Hemiptera: Cixiidae) associated with rice crops in Los Hornos, Buenos Aires, Argentina (34° 52′ S -57° 58′ W) and deposited in the Mycological Collections of the Centro de Estudios Parasitológicos y de Vectores (La Plata, Argentina), and at the USDA-Agricultural Research Service Collection of Entomopathogenic Fungal Cultures (Ithaca, New York) under the accession numbers CEP 189 and ARSEF 7776, respectively. The *M. anisopliae* strain was isolated from unidentified species of Hemiptera: Cercopidae living on *Eryngium* sp. L. (Apiaceae) plants in Esteros del Iberá, Corrientes, Argentina (28° 24′ S - 57° 07′ W) and deposited in the Mycological Collections of the Centro de Estudios Parasitológicos y de Vectores, at the USDA-Agricultural Research Service Collection of Entomopathogenic Fungal Cultures (Ithaca, New York), and at the Instituto de Botánica Carlos Spegazzini (La Plata, Argentina), under the accession numbers CEP 160, ARSEF 8376, and LPSC 908, respectively. Dr. Richard Humber, insect mycologist and curator of the USDA-Agricultural Research Service Collection of Entomopathogenic Fungal Cultures, confirmed both fungal species. The fungal isolates were maintained in culture on malt extract agar for 10 d at 25° C in darkness before being used to inoculate the planthoppers. Conidial germination was calculated for each isolate according to Lane et al. (1988).

**Figure 1.**
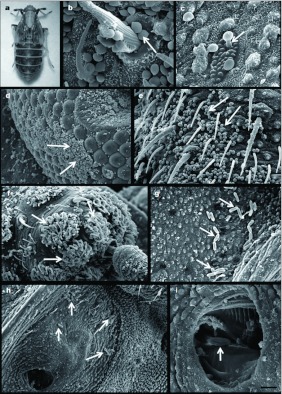
Scanning electron micrographs: (a) Photo of adult females of *Peregrinus maidis* infected with *Beauveria bassiana* CEP189 and *Metarhizium anisopliae* CEP 160, Bar: 0.7mm (b) *B. bassiana* conidia over a hair socket on the antennae (arrow), Bar: 2 ìéôé. (c) *B. bassiana* germ tube penetrating through a pore of the wax glands (arrow), Bar: 2 µm. (d) *B. bassiana* conidia between the ommatidia of the compound eye (arrows), Bar: 20 µm. (e) *B. bassiana* conidia in the second antennal segment (arrows), Bar: 10 µm. (f) *B. bassiana* conidia between the hairs of the antennal sensory pits (arrows), Bar: 20 µm. (g) *M. anisopliae* conidia near to the pores of the wax glands (arrows), Bar: 11.7 µm. (h) *M. anisopliae* conidia near the spiracle (arrows), Bar: 14 µm. (i) *M. anisopliae* conidium enclosed within the spiracle (arrow), Bar: 4 µm. High quality figures are available online.

To study the infection process with a scanning electron microscope, a total of 30 brachypterous insects (15 males and 15 females) of *P. maidis* (Figure 1a) were inoculated with each fungal isolate, and 20 insects (10 males and 10 females) were used as controls. Ten-day-old insects were inoculated in groups of 10 inside glass vials of 10cm × 1cm via topical application by spreading dry conidia with a camel-hair brush on insects. After inoculation, insects were incubated at 24 ± 1° C, at high relative humidity (>90%) and with a photoperiod of 14:10 hr (L:D), in groups of 10 in plastic Petri dishes (90mm) with filter paper moistened with sterile distilled water and conditioned with maize leaves which were removed every three days. In this experiment, at 24, 48 and 72 hr after inoculation, two males and two females inoculated with each fungal strain were fixed in 2.5% glutaraldehyde for a time period of 24 hr at 4° C, then transferred to fresh 2.5% glutaraldehyde and fixed for 45 min at room temperature, washed in 0.1M cacodylate buffer for 45 min, post-fixed in 1.0% osmium tetroxide for 1 hr at room temperature, washed in distilled water for 10 min, and dehydrated in an ascending ethanol series (10, 30, 50, 70, 80, 90, 95 and 100%) for 10 min each (adapted from Becnel 1997). Samples were dried by critical-point, coated with a gold palladium film, and examined and photographed using a JEOL 6360 LV scanning electron microscope.

To study the entomopathogenic fungi multiplication inside the body of the insect host, sets of 20 females and 20 males of *P. maidis* were inoculated with *B. bassiana* and *M. anisopliae* and incubated in the way described above. Ten males and 10 females were used as controls. Two treated insects and one control insect of each sex were fixed at 24 hr intervals for 6 days, and two insects treated of each sex were fixed at 24 and 48 hr after death. Before fixation, insects were anesthetized at -20° C for 1 min, and their wings and legs were extirpated. Treated and control insects were fixed for 24 hr in formaldehyde phosphate buffer 10% (pH 6.8; Humason 1962). After fixation, the specimens were dehydrated in a graded series (30, 50 and 75%) of ethanol and butyl alcohol, and embedded in Paraplast®. Sagittal serial sections (5–6 µm in thickness) were prepared and stained using Masson trichromic (Masson 1928) and Grocott methenamine-silver nitrate method for fungi (Grocott 1955). The stained preparations were mounted in natural Canadian balsam, and then slide preparations were observed under an Olympus microscope (CH 30).

## Results and Discussion

### Histopathology: Conidial adhesion, germination and penetration through the insect cuticle.

**Figure 2.**
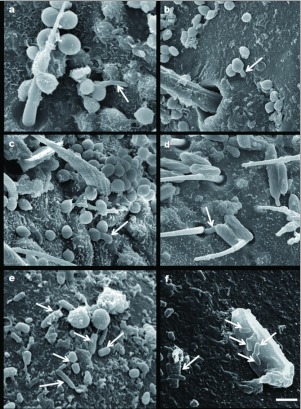
Scanning electron micrographs of adult females of *Peregrinus maidis* infected with *Beauveria bassiana* CEP189 and *Metarhizium anisopliae* CEP 160: (a–c) *B. bassiana* germ tubes (arrows) penetrating through the cuticle, Bar: 1.5 µm; 2.8 µm and 2 µm, respectively, (d) *M. anisopliae* germ tube (arrow) entering through the hair sockets situated on forewing venation, Bar: 3.3 µm. (e) Bacillus-like bacteria (arrows) associated with two globose *B. bassiana* conidia on cuticle surface, Bar: 1.7 µm. (f) Bacillus-like bacteria (arrows) associated with *M. anisopliae* conidium on cuticle surface, Bar: 1 µm. High quality figures are available online.

Under scanning electron microscopy, *B. bassiana* conidia (95.5% *in vitro* germination) were observed at high density mainly in the sternal region of the abdomen, where these were deposited in areas near to the hairs (Figure 1b) and the pores of the wax glands (Figure 1c). Conidia were also observed between the ommatidia of the compound eye (Figure 1d), in the second antennal segment, between the hairs of the sensory pits (Figure 1e–f), and at the articulating membranes of legs. *M. anisopliae* conidia (100%) *in vitro* germination) were observed in the same regions of the host insect as *B. bassiana*, but in smaller concentrations (Figure 1g). *M. anisopliae* conidia were gathered near and enclosed within the spiracles (Figure 1h–i). The hydrophobic conidia of both fungal species were able to attach to all body regions, with a preference for surfaces containing hairs, as was reported by Boucias et al. (1988). *B. bassiana* conidia were found especially to be trapped by and tightly bound to these hairs (Figure 1b), as was also previously reported by Boucias and Pendland (1991). Few germinated conidia were observed at 24, 48, and 72 hr post-inoculation on the whole body surface. For both fungal species, germ tubes that registered at 24 and 48 hr were short, and they were observed penetrating directly through the host cuticle in regions near the hairs of the second antennal segment and on the laterosternites of abdomen. After 72 hr, long and errant germ tubes were detected on the cuticular surface. In all cases, *M. anisopliae* emitted only one germ tube from each conidium, as Schabel (1978) also reported. Neither the bipolar germination nor the appressoria formation observed by Mc Cauley et al. (1968) and Vestergaard et al. (1999) were detected. The most frequent method of penetration was through the cuticle (particularly for *B. bassiana*) (Figure 1c and Figure 2 a–c), although *M. anisopliae* germ tubes were observed entering through the hair sockets situated on the forewing venation (Figure 2d). Mc Cauley (1968) reported the penetration of *M. anisopliae* though the solid cuticle as the most common method of entering the body cavity of Elaterid larvae, after the spiracles and pores of the sense organs. The preferential penetration sites in *F. occidentalis* observed by Vestergaard et al. (1999) were noted on the head, thorax, abdomen, and on the thickest part of the wings, where the conidia penetrated directly through the cuticle. *B. bassiana* conidia were able to penetrate directly through the integument, as well as through the respiratory system (Pekrul and Grula 1979). Germination on the cuticular surface was observed at 24 hr postinoculation, but the germination percentages were low (confronted with 95.5% and 100% *in vitro* for *B. bassiana* and *M. anisopliae* respectively). In the study carried out by Mc Cauley et al. (1968) most of *M. anisopliae* conidia inoculated in Elateridae (Coleoptera) larvae germinated within 24 to 48 hr after inoculation; Vestergaard et al. (1999) obtained 100%) germination of *M. anisopliae* conidia at 24 hr post-inoculation of *Frankliniella occidentalis* (Thysanoptera: Thripidae), while the results obtained by Schabel (1978) showed that germination of the same fungal species on the cuticular surface of *Hylobius pales* (Coleoptera: Curculionidae) was within 35 to 132 hr post-inoculation. For some systems, the failure of fungi to invade the insect cuticle has been attributed to the presence of inhibitory compounds such as phenols, quinones, and lipids on the cuticle surface (Smith et al. 1981; Kerwin 1984; St Leger 1991; Lord and Howard 2004). While Hubner (1958), Walstad et al. (1970) and Schabel (1978) also suggest the existence of antibiosis by the microbiota (*e.g*. other fungi and bacteria) living on the cuticular surface of the host. Similarly, bacillus-like bacteria were present in the cuticular surface of *P. maidis* treated with *B. bassiana* and *M. anisopliae* (Figure 2e–f). In recent studies, 160 bacteria strains isolated from the cuticular surface of adults of *Delphacodes kuscheli* (Hemiptera: Delphacidae) and *Dalbulus maidis* (Hemiptera: Cicadellidae) were characterized. Almost half (45.8%) of these bacteria were Gram (+) spore-forming bacilli (Toledo et al., unpublished data). The ability of some sporeforming bacteria to inhibit different species of fungi by secreting antibiotics with antifungal properties, such as iturins, subtilins, mycosubtilins, megacins, and circulins, has been well documented (Holland 1961; Katz and Demain 1977; Alippi et al. 2000)

**Figure 3.**
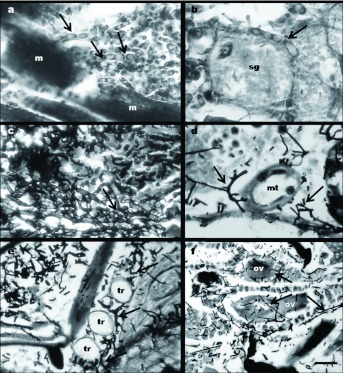
Sagittal sections of *Peregrinus maidis* infected with *Beauveria bassiana* CEP189 and *Metarhizium anisopliae* CEP 160. (a) Hyphal bodies of *M. anisopliae* (arrows) in the muscle tissue (m), Bar: 8 µm. (b) Hyphal bodies of *M. anisopliae* (arrow) in the salivary glands (sg), Bar: 4.2 µm. (c) Hyphal bodies of *B. bassiana* (arrow) inside the thorax, Bar: 7 µm. (d) *B. bassiana* hyphae (arrows) near the Malpighian tubules (mt), Bar: 7.5 µm. (e) *B. bassiana* hyphae (arrows) near the tracheae (tr), Bar: 7.5 µm. (f) *B. bassiana* hyphae (arrows) penetrating through the ovariole wall (ov), Bar: 15 µm. High quality figures are available online.

### Histopathology: Entomopathogenic fungi multiplication inside the body host

In the histological sections observed under light microscopy, Masson trichromic was the most useful stain because it permitted identification of every tissue of the insect host, whereas the Grocott coloration was useful for distinguishing the hyphae and hyphal bodies, which were stained brown and black in contrast to the green coloration of the host tissues. In the colonization events observed in this study, the formation and multiplication of hyphal bodies by both fungal species inside the host body was noted. Hyphal bodies were multiplied by the budding of pre-existent cells in accordance with Madelin (1963).

Short hyphal bodies of *M. anisopliae* were observed inside the abdomen and thorax of males and females of *P. maidis* at 4 days postinoculation (Figure 3a–b). Within 5 days post treatment, short hyphae (2.48 µm width), were recorded at the hemocoel; in some cases hyphae branched and grew near the Malpighian tubules. At 6 days postinoculation, the entire body was invaded by hyphae. Body fat was the most affected tissue. The hyphal bodies ranged between 1 and 4 µm in diameter, according to the tissue that was invaded (*i.e*. tegument 3 µm, body fat and muscle 4 µm, salivary glands 2.5 µm, lumen of the digestive tract and reproductive cavities 1–1.5 µm) (*n* = 30 for each observation). Variation in length, form and vacuolation of hyphal bodies were greatest in the early stages of development. Mc Cauley (1968) reported this same variation and assumed it was due to differences in quantity and quality of the nutrients available to the fungus. A limitation of the availability of nutrients may explain why hyphae growth within the lumen of the digestive tract, Malpighian tubules, and tracheae exhibited a distinctly smaller diameter than those just outside these systems. Hyphal bodies in the muscle tissue (Figure 3a) and in the cephalic region near the ocular peduncle contained big vacuoles (2.4–4 µm, *n* = 30). At 6 days post-inoculation, hyphae were observed inside the cavities of the reproductive system and the Malpighian tubules. At 24 hr after death, hyphae invasion of the host tissues was limited to the body fat, the muscle tissue of the thorax, and the ventral nerve cord. At 48 hr after death, hyphae and hyphal bodies were observed in all the tissues, including the oocytes inside the ovarioles in females.

Hyphal bodies of *B. bassiana* were observed inside the abdomen and thorax of *P. maidis* 5 days post-inoculation (Figure 3b). Short septated hyphae were noted in the hemocoel of males and females (3–16.5 µm, *n* = 30). In both sexes, the highest concentration of hyphae was detected in the terminal region of the abdomen between the body fat cells. In females, some elongated hyphae were observed in the muscle tissue of the thorax. At 24 hr after death, hyphal bodies were observed all over the males. These hyphae and hyphal bodies were found in all the tissues, in the cavities of the digestive system, and near the Malpighian tubules and the tracheae (Figure 3d–e). At 24 hr after death, hyphae penetrating through the ovariole wall (Figure 3f) and near the tracheae were observed in the females. At 48 hr after death, the entire abdomen and the reproductive system were invaded and the muscular tissue surrounding the hyphae was under lysis. The hyphal bodies ranged between 1 and 2.8 µm in diameter (*n* = 30). Neither penetration pegs nor cellular defense reactions associated with the hyphal bodies were observed in any of the saggital sections studied.

Due to the scarce number of germinated conidia observed on the insects' cuticular surface, it is likely that the bacteria over the external cuticle is interacting with fungal conidia, playing a role of antibiosis that will not allow the fungal pathogens to germinate and penetrate as suggested in the literature (Hubner 1958; Walstad et al. 1970; Schabel 1978). The *in vitro* antagonistic effect of the bacteria isolated from delphacids and cicadellids against the growth of *B. bassiana* was recently studied. This included evaluating 160 strains and recording inhibition percentages ranging between 0% and 83% (Toledo et al, unpublished data). In addition, the high quantity of wax that usually covers the cuticle of these insects could have some chemical compounds that might inhibit further germination of fungal conidia. Either or both of these scenarios could be occurring as an antagonistic and inhibitory mechanism, and further research is necessary to test this hypothesis.
